# Continuous wavelet transform-based analysis of shoulder muscle fatigue during dynamic abduction: a surface electromyography study

**DOI:** 10.3389/fbioe.2026.1855236

**Published:** 2026-07-23

**Authors:** Jonathan Sommer, Richard Julius Freytag, Louisa-Marie Lütz, Jan-Marek Meyer, Mika Jan Milobara, Mahmoud Ragab, Kristian Welle, Dieter Christian Wirtz, Davide Cucchi

**Affiliations:** Department of Orthopedics and Traumatology, University Hospital Bonn, Bonn, Germany

**Keywords:** continuous wavelet transform, dynamic movement analysis, muscle fatigue, shoulder muscles, surface electromyography

## Abstract

**Background:**

Quantifying neuromuscular fatigue from surface electromyography (sEMG) signals during dynamic movements remains a methodological challenge due to the signals’ non-stationarity. While wavelet-based approaches have demonstrated potential for analyzing muscle fatigue during dynamic tasks, substantial heterogeneity exists regarding movement protocols, signal-processing strategies, and fatigue metrics. Given the shoulders’ central role in daily activities, this study evaluates a predefined Continuous Wavelet Transform (CWT)-based analysis framework for assessing fatigue-related spectral changes during dynamic shoulder abduction.

**Methods:**

Eight healthy subjects performed eleven standardized metronome-guided shoulder abductions under two conditions: without (W_0_) and with (W_1_) 2-kg handheld weights on three consecutive days. Bilateral sEMG recordings from the upper trapezius (UT) and lower trapezius (LT) were analyzed using CWT-based median frequency analysis. For comparison, the complete analytical pipeline was additionally applied using FFT-based median frequency estimation. Signal preprocessing included Butterworth- and Bandnotch filtering. After signal division into 50%-overlapping 1-s time windows, fatigue-related spectral changes were quantified by linear regression of median frequency over time. Statistical analysis was performed using repeated-measures ANOVA.

**Results:**

Repeated-measures ANOVA revealed significant load-related reductions in median-frequency regression slopes for the right UT (F (1,7) = 10.09, p = 0.016, ηp^2^ = 0.59), left UT (F (1,7) = 15.71, p = 0.006, ηp^2^ = 0.69), and right LT (F (1,7) = 7.54, p = 0.029, ηp^2^ = 0.52). Similar trends were observed in the left LT but did not reach statistical significance. Analysis of muscular differences in response to load revealed significantly greater responses in the UT compared with the LT (F (1,15) = 6.35, p = 0.024, ηp^2^ = 0.3). Exploratory hand dominance analysis demonstrated larger slope reductions on the non-dominant side, reaching statistical significance only for the LT in W_1_ (p = 0.037). Additional FFT analysis showed comparable fatigue-related trends but consistently weaker statistical evidence than the CWT-based approach.

**Conclusion:**

These findings demonstrate the feasibility of the applied CWT-based framework for detecting fatigue-related spectral changes in dynamic shoulder movements. By addressing limitations of conventional frequency-domain approaches, the approach represents a promising framework for functional sEMG analysis and may support a basis for future applications in biomechanics, rehabilitation, and performance monitoring.

## 1 Introduction

Quantitative assessment of neuromuscular fatigue provides valuable insights into muscular function and has broad relevance in biomechanics and rehabilitation engineering. Understanding fatigue-related dynamics enables the development of innovative diagnostic and monitoring tools, biofeedback systems, and individualized rehabilitation strategies. Moreover, identifying muscular imbalances and dysfunctions can reduce the risk of both primary and secondary injuries by informing targeted training and rehabilitation interventions (Alba-Jiménez et al., 2022; Croisier, 2004; Muñoz-Gracia et al., 2025). However, current analytical approaches often fail to capture the dynamic nature of movements, limiting their applicability in these contexts.

Several techniques have been proposed to measure fatigue (Al-Mulla et al., 2011), including biochemical markers such as lactate (Okano et al., 2025), blood flow monitoring via near-infrared spectroscopy (Corral-Pérez et al., 2024), and mechanomyography (Benwali et al., 2022; Islam et al., 2013). Among these approaches, surface electromyography (sEMG) is particularly well-established, offering non-invasive, real-time monitoring of myoelectric manifestations of fatigue (Al-Mulla et al., 2011). These changes can be analyzed across different analytical domains (Sun et al., 2022).

In the time domain, fatigue typically manifests as amplitude-based increases in root-mean-square (RMS) and integrated EMG (iEMG) values, reflecting compensatory motor-unit recruitment. Additional markers such as zero-crossing rates have also been reported (Cui et al., 2021; Kim et al., 2018). Frequency-domain analysis generally shows a shift of the power spectrum towards lower frequencies, quantified by parameters such as mean and median frequencies. The power spectrum is usually calculated via the Fast Fourier Transform (FFT) (Teufl et al., 2021). While FFT-based methods are well-suited for static or isometric contractions, they are limited in capturing the dynamics of non-stationary signals during functional, dynamic movements.

Time-frequency methods, including the Short-Time Fourier Transform (STFT), Continuous Wavelet Transform (CWT), Discrete Wavelet Transform (DWT), and Wigner-Ville distribution (WVD), have been introduced to address these limitations. Comparative studies indicate that the Continuous Wavelet Transform (CWT) provides superior temporal and spectral resolutions compared to the STFT, FFT, and WVD (Karlsson et al., 2000), with results comparable to those of the DWT but faster computational performance (Abdullah et al., 2011). The choice of mother wavelet critically influences analysis robustness and interpretability, with the Morlet wavelet commonly favored for its high time-frequency resolution in non-stationary signal analysis (Silik et al., 2021; Ergen et al., 2012).

Wavelet-based approaches have been explored for assessing fatigue-related spectral changes across a variety of dynamic movement contexts. Recent studies have successfully applied wavelet analysis to rowing exercises, upper-extremity movements, and other functional tasks, highlighting the potential of time-frequency methods for dynamic sEMG analysis (Daniel and Małachowski, 2023; Daniel et al., 2024; Mota-Carmona et al., 2022). Nevertheless, substantial methodological heterogeneity exists across the literature with respect to movement protocols, preprocessing strategies, wavelet implementations as well as analysis parameters. As a result, direct comparison between studies remains challenging, and methodological variability continues to limit the transferability of findings across different experimental settings.

Given the central role of the shoulder in daily occupational and athletic activities, as well as its importance in clinical decision-making and rehabilitation (Wenning et al., 2023; Wenning et al., 2024), there is a need for robust analytical approaches capable of capturing shoulder and periscapular muscle fatigue under non-stationary conditions. Improved characterization of these adaptations may contribute to objective monitoring of rehabilitation progress, identification of neuromuscular dysfunction, and optimization of exercise-based interventions.

Therefore, the present proof-of-concept study applies and evaluates a predefined CWT-based analysis framework for assessing fatigue-related spectral changes during dynamic shoulder abduction using sEMG signals. By examining load-dependent changes in the upper and lower trapezius muscles across repeated measurements, this study aims to evaluate the feasibility of the proposed framework for assessing fatigue-related spectral changes during dynamic shoulder movement analysis, thereby improving methodological consistency in this field.

## 2 Materials and methods

### 2.1 Participants

Eight healthy male participants without any known scapulothoracic pathologies were enrolled and followed a standardized movement protocol over three consecutive days. The mean age of the participants was 24.75 ± 3.85 years (range: 22–34 years). The mean BMI was 25.29 ± 1.93 kg/m^2^. Seven participants were right-handed, whereas one was left-handed. All participants were recreationally active and able to complete the study protocol without limitations. Exclusion criteria included fractures of the clavicle or the humerus, shoulder or acromioclavicular joint dislocations, rotator cuff tears, or any other muscle-related injuries affecting either shoulder. To ensure the feasibility of the movement protocol, participants were required to be in good physical condition and capable of performing repeated upper-limb movements with 2 kg dumbbells.

### 2.2 Data collection

Each participant performed 11 shoulder abduction cycles, each lasting 6 s, guided by a metronome set to 60 beats per minute. Starting from a neutral position, participants performed simultaneous bilateral abductions to 180° within 3 s, then returned to the starting position within the following 3 s. Prior to testing, participants were familiarized with a fluent movement sequence through a demonstration and up to 3 example sequences. Each run included an initial 5-s rest and a final 5-s pause to facilitate metronome synchronization. Exercises were initially performed without additional weight (W_0_) and subsequently with 2 kg dumbbells (W_1_), separated by a standardized two-minute recovery phase (McDonald et al., 2017). The entire protocol was repeated over three consecutive days.

#### 2.2.1 Electrode placement

**FIGURE 1:**
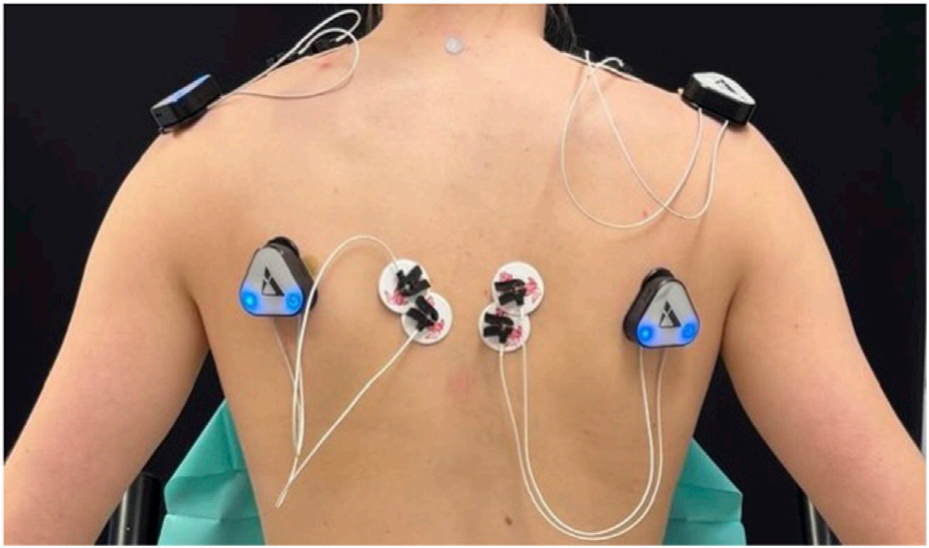
Electrode and sensor placement.

sEMG signals were recorded from the upper trapezius (UT) and lower trapezius (LT) following SENIAM (Surface Electromyography for the non-invasive assessment of muscles) guidelines (Figure 1). Both muscles were selected for their key roles in scapulothoracic control during shoulder abduction. Muscular imbalances between UT and LT are associated with common shoulder pathologies, such as chronic neck pain (Wegner et al., 2010) and symptomatic scapulothoracic dyskinesis (Javdaneh et al., 2025), potentially reducing athletic performance (Costa E Silva Cabral et al., 2024). Their accessibility for sEMG results in minimized signal noise interference and high reproducibility (Cid et al., 2018).

To allow adequate data quality, the skin was shaved when necessary and degreased prior to electrode placement (Sousa et al., 2023). For the LT, two electrodes were placed parallel to the muscle fibres at one-third of the distance between the 8th thoracic vertebra and the scapular trigonum spinae. For the UT, two electrodes were placed in parallel at the midpoint of the line connecting the spinous process of the 7th cervical vertebra and the acromion. Reference electrodes attached to the UT were placed on the acromion, and for the LT on the inferior angle of the scapula. Wireless sensors collecting the sEMG data from the specific muscle were directly affixed to the reference electrode.

sEMG-signals were recorded using the wireless iEMG system (DIERS International GmbH, Germany) with a sampling frequency of 2000 Hz. Disposable pre-gelled Ag/AgCl surface electrodes (Dahlhausen Medizintechnik GmbH, Germany) with a total diameter of 35 mm were used for signal acquisition. Electrodes were placed in a bipolar configuration with an inter-electrode distance of 20 mm following SENIAM recommendations.

### 2.3 Data processing and analysis

The signal processing and data analysis were conducted in MATLAB Version R2023a. The preprocessing pipeline consisted of three sequential steps. First, raw sEMG signals were filtered using a 4^th^-order Butterworth bandpass filter between 20 Hz and 500 Hz. This frequency range was selected in accordance with commonly applied sEMG preprocessing approaches to preserve physiologically relevant signal information while attenuating low-frequency motion artifacts and high-frequency noise (Boyer et al., 2023). Secondly, a notch filter with a quality factor Q = 35 at 50 Hz was used to remove power line interference, following the recommendations of Boyer et al. (2023). Finally, the first and last 5 seconds of each trial were removed to exclude resting periods before and after the movement task, ensuring that only active movement phase was included in the subsequent analysis.

The filtered signal was segmented into 1-s time windows with 50% overlap. The window length was selected based on previous sEMG studies reporting an improved balance between estimate stability and temporal resolution when compared with substantially shorter analysis windows (Burden et al., 2014). A 50% overlap was applied to improve temporal continuity between adjacent estimates and to provide smoother tracking of spectral changes over time (De La Fuente et al., 2021). Each analysis window was analyzed via the CWT using MATLAB’s built-in CWT function (R2023a). The analytic Morlet wavelet (amor) was selected because of its favorable time-frequency localization properties and its widespread use in the analysis of non-stationary biomedical signals (Ergen et al., 2012; Silik et al., 2021). 10 voices per octave were used, covering a frequency range of 2.88–736.57 Hz across 8 octaves. The effective analysis range was constrained to 20–500 Hz by the preceding bandpass filter. Spectral energy per frequency was calculated as the squared magnitude of the wavelet coefficients. The median frequency was defined as the frequency at which the cumulative energy reaches 50% of the total energy in the specific window and was calculated via the following formula:

$$\int_{f=0}^{fmed}P\left(f\right)df=\frac{1}{2}\int_{f=0}^{fmax}P\left(f\right)df$$

Where:

- *P (f)* is the power spectral density at frequency *f*
- *fmed* is the median frequency
- *fmax* is the maximum frequency considered

**FIGURE 2:**
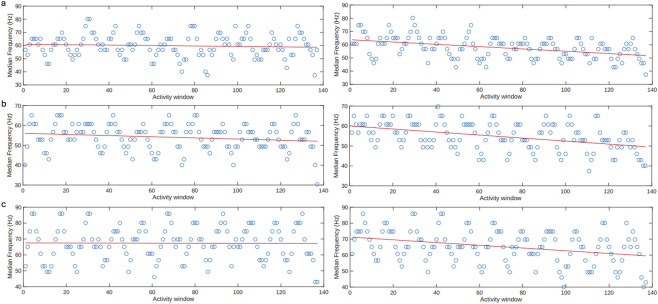
Representative examples of the CWT-based median-frequency analysis for the right UT during dynamic shoulder abduction. Panels **(a–c)** show data from three different participants. While W0 is displayed on the left, W1 is displayed on the right.

Median frequency values were computed for each analysis window. Fatigue was quantified using linear regression analysis of the CWT-derived median frequency trend over time. The linear regression was defined as fitting a linear function to the median frequency values of the analysis windows, minimizing squared residuals (Figure 2). The slope of this regression was used as the primary outcome, with negative slopes indicating muscle fatigue (Corvini and Conforto, 2022; Daniel et al., 2024).

### 2.4 Data analysis

The algorithm was evaluated in four complementary ways:

- Muscle fatigue trends under load: Comparing median frequency slopes between no-load (W_0_) and 2-kg load (W_1_) conditions
- Intermuscular differences: Comparing UT and LT to assess sensitivity to functional roles
- Hand dominance: Comparing dominant versus non-dominant sides to detect potential differences
- Comparison with a conventional frequency-domain approach: The complete methodological framework was additionally applied using FFT-based median frequency estimation. Signal preprocessing, window segmentation, median frequency calculation, linear regression analysis, and statistical evaluation were performed identically to the CWT-based workflow. For the FFT analysis, a Hamming window was applied prior to spectral estimation to reduce spectral leakage before calculating the median frequency

### 2.5 Statistical analysis

All statistical analyses were performed using IBM SPSS Statistics (Version 30.0.0, IBM Corp., Armonk, NY, United States). Statistical significance was set at p < 0.05. To investigate load-induced fatigue, repeated-measures ANOVA (RM-ANOVA) was performed with day and load as within-subject factors. To assess intermuscular differences in the response to external load, load-induced changes in median-frequency regression slopes were calculated for each measurement day as the difference between loaded and unloaded conditions for each muscle-side combination. The differences were subsequently analyzed using RM-ANOVA with day and muscle as within-subject factors. The effect of hand dominance was evaluated using RM-ANOVA with day and hand dominance as within-subject factors. Normality was assessed using the Shapiro-Wilk test prior to analysis, and no significant deviations from normality were detected. Sphericity was evaluated using Mauchly’s test for within-subject factors with more than two levels. When the assumption of sphericity was violated, Greenhouse-Geisser-corrected results were reported. Effect sizes were expressed as partial eta squared (ηp^2^) with values of 0.01, 0.06, and 0.14 representing small, medium, and large effects. The same statistical workflow was subsequently applied to the FFT-derived regression slopes to enable direct comparison between both CWT- and FFT-based analytical approaches.

## 3 Results

In total, 192 sEMG signals from the eight included participants were successfully analyzed (Figure 2). No technical difficulties were encountered, and the analysis protocol was successfully applied to all datasets.

### 3.1 Muscle fatigue trends under load

The CWT-based algorithm revealed systematic reductions in median-frequency regression slopes across all four muscles between W_0_ and W_1_, indicating increased fatigue-related spectral changes under external load (Table 1).

**TABLE 1:** Effect of external load on median frequency regression slopes.

Muscle	W0	W1	F (1,7)	p-value	ηp^2^
UT right	−0.0293	−0.0557	10.09	0.016	0.59
UT left	−0.0235	−0.0563	18.73	0.003	0.73
LT right	−0.0143	−0.0259	18.51	0.004	0.73
LT left	−0.0199	−0.0320	4.13	0.082	0.37

RM-ANOVA demonstrated significant load-related effects for both the right and left UT and for the right LT, while the left LT showed a comparable trend that did not reach statistical significance.

**FIGURE 3:**
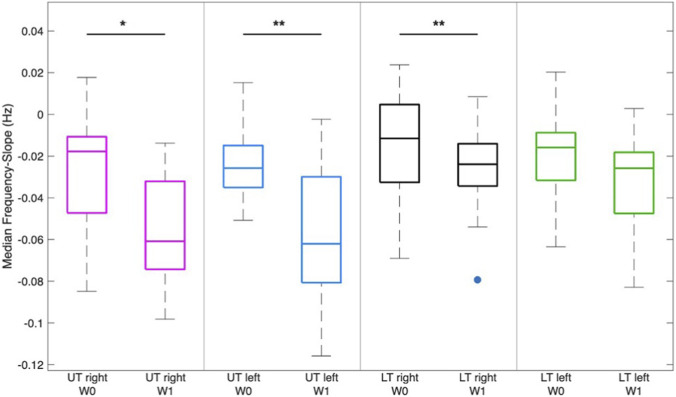
Box plot of load-induced changes in median frequency regression slopes. Each box represents the interquartile range (IQR), which contains 50% of the values. The horizontal line in each box represents the median regression slope. Whiskers denote the minimum and maximum values, while individual points represent outliers. Statistical significance is indicated as follows: * p < 0.05; ** p < 0.01; *** p < 0.001.

Among the examined muscles, the observed UT slopes were generally more pronounced than the LT slopes. While the UT showed a more substantial effect on the left side, the LT exhibited a stronger effect on the right side. Large effect sizes were observed across all analyses. Overall, load-induced reductions in median-frequency regression slopes were observed across all muscles on all sides (Figure 3).

### 3.2 Intermuscular fatigue analysis

To investigate intermuscular differences in load response, changes in median-frequency regression slopes between W0 and W1 were compared between the UT and LT (Table 2).

**TABLE 2:** Intermuscular comparison of linear regression slopes of the UT and LT, along with their distribution characteristics.

Muscle	Mean Δ (W1-W0)	F (1,15)	p-value	ηp^2^
UT	−0.0293	6.35	0.024	0.3
LT	−0.0119			

**FIGURE 4:**
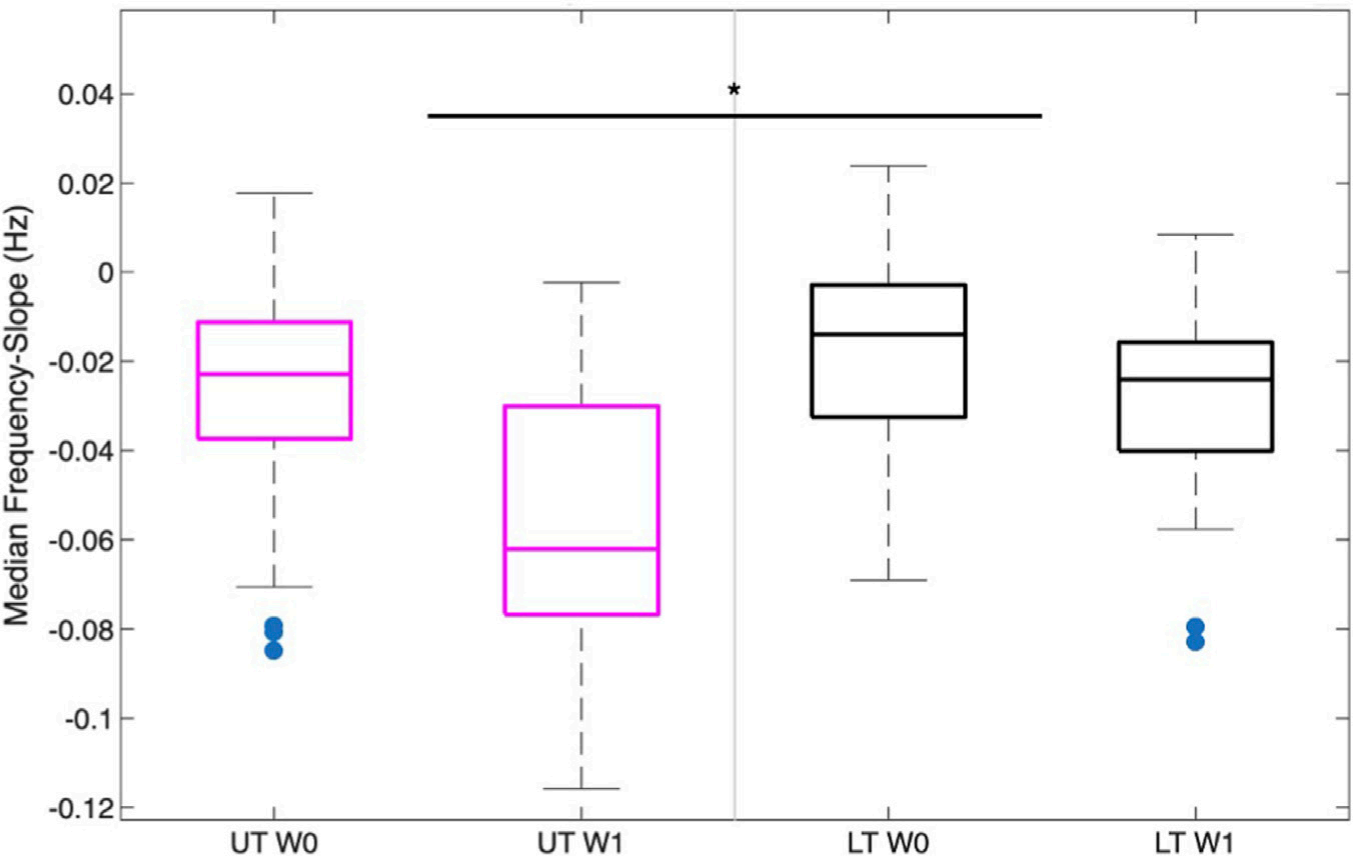
Box plot of intermuscular comparison of linear regression slopes. Each box represents the IQR, which contains 50% of the values. The horizontal line in each box represents the median regression slope. Whiskers denote the minimum and maximum values, while individual points represent outliers. Statistical significance is indicated as follows: * p < 0.05; ** p < 0.01; *** p < 0.001.

RM-ANOVA revealed a significant main effect of muscle on changes in median-frequency regression slopes, with a more pronounced decrease in UT than in LT in response to load. A Large effect size was found for the main effect of the muscle (Figure 4).

### 3.3 Muscle fatigue trends depending on hand dominance

A comparison between the dominant and non-dominant sides revealed a stronger trend of fatigue-related spectral changes on the non-dominant side (Table 3).

**TABLE 3:** Effect of hand-dominance on changes in the median frequency regression slope.

Muscle	Condition	Dominant	Non-dominant	F (1,7)	p-value	ηp^2^
UT	W0	−0.0260	−0.0268	0.02	0.881	0.003
UT	W1	−0.0542	−0.0572	0.20	0.668	0.03
LT	W0	−0.0129	−0.0212	4.61	0.069	0.40
LT	W1	−0.0248	−0.0331	6.65	0.037	0.49

For the UT, the median frequency slopes showed only slight reductions on the non-dominant side in both unloaded and loaded conditions. A RM-ANOVA indicated no significant differences between the two sides, with small effect sizes.

**FIGURE 5:**
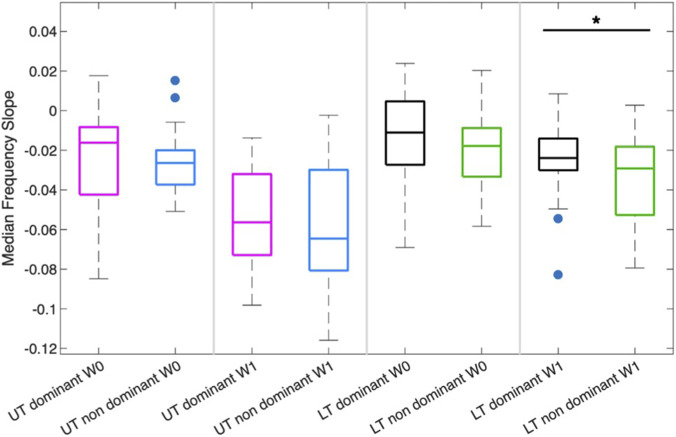
Graphical representation of the effect of hand-dominance on changes in regression slopes. Each box represents the IQR, which contains 50% of the values. The horizontal line in each box represents the median regression slope. Whiskers denote the minimum and maximum values, while individual points represent outliers.

For the LT, slope reductions were more pronounced. While the effect of dominance did not reach statistical significance in W0, a significant effect was observed in W1. Large effect sizes were observed for both LT analyses (Figure 5).

### 3.4 Comparison with conventional FFT analyses

To evaluate the relationship between the proposed CWT-based workflow and a conventional frequency-domain approach, the complete analysis pipeline was additionally applied using FFT-based median frequency estimation. Both methods demonstrated comparable load-related reductions in median frequency regression slopes (Table 4).

**TABLE 4:** Comparison of median frequency regression slopes obtained using CWT and FFT analyses.

Muscle	Method	W0	W1	F (1,7)	p-value	ηp^2^
UT right	CWT	−0.0293	−0.0557	10.09	0.016	0.59
	FFT	−0.0280	−0.0504	5.55	0.052	0.44
UT left	CWT	−0.0235	−0.0563	18.73	0.003	0.73
	FFT	−0.0209	−0.0534	18.25	0.004	0.72
LT right	CWT	−0.0143	−0.0259	18.51	0.004	0.73
	FFT	−0.0142	−0.0250	13.52	0.008	0.66
LT left	CWT	−0.0199	−0.0320	4.13	0.082	0.37
	FFT	−0.0207	−0.0306	2.47	0.160	0.26

However, the CWT-based analysis consistently yielded larger F-values, lower p-values, and larger effect sizes than the corresponding FFT-based analysis across all investigated muscles. The largest difference was observed for the right UT, where the load effect reached statistical significance using CWT but not FFT.

## 4 Discussion

The present proof-of-concept study applied a CWT-based framework to assess neuromuscular fatigue-related spectral changes in dynamic sEMG signals during dynamic shoulder abduction. External loading resulted in steeper negative median-frequency regression slopes, particularly in the UT, indicating load-dependent changes in the spectral characteristics of the sEMG signal. Furthermore, significant intermuscular differences were observed, suggesting that the framework is sensitive to different muscular responses during dynamic shoulder movements.

### 4.1 Comparison with previous literature

The present findings are consistent with previous studies demonstrating the utility of wavelet-based approaches for analyzing dynamic sEMG signals and fatigue-related spectral changes (Daniel and Małachowski, 2023; Daniel et al., 2024; Mota-Carmona et al., 2022). Similar to these investigations, load-dependent reductions in frequency-related parameters were observed, supporting the ability of time-frequency methods to characterize spectral adaptations during dynamic muscular activity. Previous dynamic fatigue studies have investigated a variety of movement tasks and muscle groups. For example, Chowdhury R. H. et al. (2013a), Chowdhury and Nimbarte, (2015) demonstrated the suitability of DWT-based approaches for assessing UT fatigue during repetitive upper-extremity tasks and reported advantages over conventional FFT analysis. Aranha et al. (2023) examined muscle fatigue of the rotator cuff in isometric contractions, whereas Schwensow et al. (2022) applied CWT analysis to upper-limb movements on a rowing ergometer. Although Schwensow et al. (2022) also included several trunk and extrinsic shoulder muscles in their analysis, an important difference from the present study lies in the movement protocol employed. Their assessment consisted of consecutive rowing bouts with progressively increasing stroke rates, offering a different, potentially less standardized approach to evaluating fatigue-related changes. Furthermore, the trapezius muscle, as a key scapular stabilizer during shoulder motion, was not included in their analysis. The present study focuses on standardized, metronome-guided cyclic shoulder abduction and applies CWT-based analysis to both the UT and LT, offering a possible advantage over the previous publications in terms of applicability in a clinical setting. Although direct comparisons are limited by differences in study design, movement protocols, and analytical methods, the overall pattern of load-dependent reductions in frequency-related parameters is consistent with the existing literature. However, considerable methodological heterogeneity exists across published studies regarding movement protocols, analyzed muscle groups, and signal-processing strategies, limiting direct comparisons between investigations (Sun et al., 2022).

To further evaluate the proposed CWT-based framework, the complete dataset was additionally analyzed using an identical FFT-based processing pipeline. Both approaches demonstrated comparable load-related fatigue patterns across all investigated muscles. However, the CWT-based analysis consistently showed larger F-values, lower p-values, and larger effect sizes than the corresponding FFT-based analyses, while the overall physiological interpretation remained unchanged. These findings are consistent with previous reports suggesting that wavelet-based approaches are more sensitive for analyzing non-stationary sEMG signals during dynamic movements, while still capturing the same underlying fatigue-related spectral adaptations as conventional FFT-based methods (Karlsson et al., 2000; Sun et al., 2022). Accordingly, the present findings should be interpreted as evidence of improved analytical sensitivity for detecting fatigue-related spectral changes within this cohort rather than as evidence that CWT reveals fundamentally different physiological or biomechanical information beyond that obtained with FFT-based analysis. Whether the time-frequency characteristics of CWT can provide additional time-localized or movement-phase-specific physiological insights remains to be determined in future studies.

Therefore, the present findings should primarily be interpreted as evidence supporting the feasibility of the applied framework for detecting fatigue-related spectral changes during dynamic shoulder abduction. While the underlying signal-processing principles are based on previously published methodologies, the present study applies them within a standardized shoulder-specific assessment protocol incorporating metronome-guided cyclic shoulder abduction, CWT analysis, and bilateral trapezius assessment. Consequently, the current work should be regarded as a methodological refinement and shoulder-specific application of existing wavelet-based fatigue assessment techniques.

### 4.2 Load-induced changes

The methodology framework consistently identified bilateral reductions in median-frequency regression slopes under load, confirming the method’s sensitivity to fatigue-related spectral changes. The UT showed greater reductions, indicating greater susceptibility to fatigue-related changes during the abduction task. Intermuscular differences were statistically significant and align with previous studies reporting substantially higher activation of the UT than of the LT during shoulder abduction (Kara et al., 2021; Ko and Oh, 2023). Similarly, Kim et al. (2022) found greater UT activation levels in healthy individuals during isometric shoulder abductions across multiple load levels. The more pronounced spectral changes observed in the UT, therefore, appear compatible with previously described differences in muscle activation patterns during shoulder abduction. However, the present study design does not allow direct conclusions regarding the physiological mechanisms underlying the observed intermuscular differences. In particular, it cannot be determined whether the larger spectral changes observed in the UT reflect greater muscular loading, greater susceptibility to fatigue-related spectral changes, or a combination of both. Consequently, the present findings should be interpreted as evidence of different spectral responses within the study cohort between the two muscles rather than as direct evidence of specific biomechanical or physiological mechanisms. Taken together, the results suggest that the proposed CWT-based framework can detect muscle-specific responses to external loading during dynamic shoulder abduction and may therefore provide useful information on spectral differences between muscles during dynamic movements.

### 4.3 Hand dominance

Exploratory analysis of the role of hand dominance on muscle fatigue in dynamic exercises indicated a stronger trend towards muscle fatigue on the non-dominant side, although statistical significance was only reached for the LT in W1. However, observed effect sizes suggest potentially meaningful asymmetries. Previous research has demonstrated asymmetries related to hand-dominance, including differences in strength and fatigue development (Bagesteiro and Sainburg, 2002; Farina et al., 2003; Foley et al., 2025). The direction of the observed trends is therefore generally consistent with the existing literature. However, the study cohort consisted of only seven right-handed participants and one left-handed participant, resulting in a substantial imbalance between dominance groups, reflecting that of the general population. Consequently, hand dominance was largely confounded with body side, limiting the ability to distinguish true dominance-related effects from potential side-specific differences. Therefore, the present findings should be regarded as exploratory rather than as evidence of a genuine effect of hand dominance on fatigue-related spectral changes during dynamic shoulder movements.

### 4.4 Result variability

While most trials demonstrated the expected decrease in median frequency over time, isolated cases of positive regression slopes were observed. These atypical responses deviate from classical fatigue-related spectral shifts and highlight the inherent variability of sEMG signals, particularly under dynamic conditions. Several physiological and methodological factors may explain these findings. Increased external load can recruit higher-threshold motor units with higher conduction velocities, which may shift the power spectrum toward higher frequencies independently of fatigue-related physiological processes (Škarabot et al., 2023). In addition, dynamic movement introduces non-stationary conditions, including variations in muscle length, contraction velocity, and electrode displacement, all of which can influence spectral estimates.

Signal variability is further affected by factors such as electrode placement, crosstalk, and skin preparation, which are well-known sources of variability in sEMG measurements (Hermens et al., 2000). The relatively modest preprocessing applied in this study, limited to Butterworth bandpass and notch filtering (De Luca et al., 2010), was deliberately chosen to preserve relevant signal information. While this may have contributed to result variability, extensive preprocessing risks filtering out relevant raw signal information that can introduce bias and potentially remove physiologically meaningful information (Chowdhury S. K. et al., 2013b; De Luca et al., 2010). Overall, these findings emphasize the complexity of interpreting frequency-based fatigue metrics in dynamic tasks and underline the importance of robust time-frequency approaches such as the CWT.

### 4.5 Limitations and future directions

Some limitations should be considered when interpreting the present findings. First, as a proof-of-concept study, the sample size was small (n = 8), limiting both statistical power and the generalizability of the results. However, this approach and the chosen sample size are consistent with previous methodological studies aiming to establish the feasibility of novel fatigue-analysis workflows before extending investigations to larger and clinical populations (Daniel et al., 2024; Mota-Carmona et al., 2022). Second, the study cohort consisted exclusively of healthy young male participants. This decision was made to reduce potential variability related to sex-specific differences in muscle activation patterns (Bikram Panday et al., 2025; Nuzzo, 2023) and thereby increase internal consistency. Nevertheless, the findings cannot be generalized to female participants, older individuals, or clinical populations. Third, the external load applied in the W1 condition was fixed at 2 kg and was not normalized to individual strength levels. Consequently, the relative effort required to perform the task varied among participants. Fourth, the W0 and W1 conditions were performed in a fixed sequence. Although a standardized 2 -min recovery interval was provided between conditions, no physiological measurements were obtained to verify complete recovery. Therefore, residual fatigue effects and potential order-related bias cannot be excluded. However, the choice was made in accordance with McDonald et al. (2017), who demonstrated that a two-minute rest interval between maximal exertions allowed participants to complete at least 48 efforts without signs of myoelectric fatigue. Finally, no independent physiological markers of fatigue were collected. Consequently, the observed findings should be interpreted as fatigue-related spectral changes rather than direct confirmation of physiological fatigue.

Future studies could overcome these limitations by evaluating the proposed framework in larger and more diverse cohorts, including female participants and clinical populations. In addition, further investigations could incorporate independent physiological fatigue measures, such as maximum voluntary contraction, perceived exertion ratings, or heart rate monitoring, to further validate the relationship between fatigue-related spectral changes and physiological fatigue. Furthermore, the influence of methodological parameters, including window length and overlap settings, could also be investigated.

## 5 Conclusion

This proof-of-concept study demonstrates the feasibility of the applied CWT-based framework for detecting fatigue-related spectral changes in sEMG signals during dynamic shoulder abduction. The proposed method demonstrated sensitivity to load-dependent decreases in median-frequency regression slopes in the upper and lower trapezius and enabled differentiation of intermuscular responses between the upper and lower trapezius muscles. Compared with an equivalent FFT-based analysis, the proposed framework suggests improved sensitivity for detecting fatigue-related spectral changes while yielding the same overall physiological interpretation of load-dependent median-frequency changes. Thus, the present work offers a standardized CWT-based analytical framework for dynamic shoulder sEMG analysis and provides a foundation for future methodological and clinical investigations.

## Data Availability

The raw data supporting the conclusions of this article will be made available by the authors, without undue reservation.
